# Exposure to psychotropic drugs before and during pregnancy: what has changed over the last two decades?

**DOI:** 10.1007/s00737-023-01290-8

**Published:** 2023-01-14

**Authors:** Robiyanto Robiyanto, Catharina C M Schuiling-Veninga, Jens H J Bos, Eelko Hak, Eugène P van Puijenbroek

**Affiliations:** 1 Unit of Pharmacotherapy, -Epidemiology, & -Economics, Groningen Research Institute of Pharmacy, University of Groningen, Groningen, The Netherlands; 2 Program Studi Farmasi, Fakultas Kedokteran, Universitas Tanjungpura, Pontianak, Indonesia; 3 Netherlands Pharmacovigilance Centre Lareb, ‘s-Hertogenbosch, The Netherlands

**Keywords:** Exposure rate, Acceptance rate, Pregnancy, Psychotropic drugs

## Abstract

Trends in prescribing psychotropic drugs before and during pregnancy may have changed over the years, but actual information is lacking. We therefore compared and assessed the exposure and acceptance rates of classes of antipsychotic (+ lithium), anxiolytic, sedative/hypnotic, antidepressant, and psychostimulant before and during pregnancy in the past two decades. All singleton pregnancies with ≥1 prescription of psychotropic drug from six months before pregnancy until child’s birthdate were identified in the pregnancy subset of the IADB.nl prescription database. The prescription patterns of psychotropics were distinguished as continuation rate (CR), initiation rate (IR), discontinuation rate (DR), total exposure rate (TER), and acceptance rate. Singleton pregnancies exposed to psychotropic drugs before and during pregnancy increased from 118.4 to 136.5 (per 1000 singleton pregnancies) between decades. Changing trends were observed in decade 2, including a high increase in the TER of antipsychotic class (3.3 to 6.8) and antidepressant class (23.0 to 40.6). A marked increase for individual drugs was seen for sertraline (TER: 0.6 to 6.6 and PAT: 35.3% to 82.5%), citalopram (TER: 2.3 to 10.0 and PAT: 51.1% to 74.6%), and quetiapine (TER: 0.4 to 3.1 and PAT: 57.1% to 66.0%). Although the total exposure rates of five classes of psychotropics in singleton pregnancies increased in decade 2, only antidepressant class had a higher acceptance rate during pregnancy. Certain SSRI antidepressants and atypical antipsychotics were more frequently prescribed in decade 2 than in decade 1, reflecting that treatment options were preferred for safer treatment choices.

**Supplementary Information:**

The online version contains supplementary material available at 10.1007/s00737-023-01290-8.

## Introduction

Psychotropic medications, like antipsychotics, anxiolytics, sedatives/hypnotics, and antidepressants, are among the most frequently prescribed drugs to pregnant women (Schirm et al. 2004; Bakker et al. 2006; Houben et al. 2020). Between 2006 and 2011, over 10% of pregnant women in the US received at least one psychotropic drug (Hanley and Mintzes 2014). The prescription rate of antipsychotics and antidepressants during pregnancy in the Netherlands from 1994 to 2003 ranged from 0.09–0.19 (per 1000 women), and for anxiolytics and sedatives/hypnotics, the range was 0.09–0.15 (Bakker et al. 2006). Despite the use of some psychotropic drugs during pregnancy had been reported to increase in the past years by previous studies, updated information on how prescribing trend of psychotropics in the last recent years in pregnancy population may have changed is still lacking.

In some cases, pregnancy problems may induce stress or affect pregnant women’s mental condition, urging them to continue the treatment they used before pregnancy. Ideally, when planning for a pregnancy program, a woman should inform her healthcare provider if she has a history of using psychotropic medication before pregnancy. Since knowledge about the safe use of psychotropic drugs during pregnancy is essential yet limited, it is important to balance the potential risks and benefits for the mother and unborn child when deciding to continue, initiate, or stop a psychotropic treatment when a woman is pregnant. This decision should consider the severity of psychiatric illnesses (including risk of relapse), previous treatment response, and patient’s preference. The safest and effective maternal dose of medication should be prescribed during pregnancy if the benefits of prescribing a psychotropic drug outweigh its potential risk (Orsolini and Bellantuono 2022). Monitoring trends in drug utilization is required to evaluate whether, in clinical practice, the choice of psychotropic medication is appropriate, safe, and in line with current guidelines for pregnant women.

In this study, we aimed to describe and analyze the clinical practice of prescribing patterns of antipsychotics, anxiolytics, sedatives/hypnotics, antidepressants, and psychostimulants among Dutch pregnant women before and during pregnancy by looking at their exposure and acceptance rates over the last two decades (2000–2019).

## Methods

### Study design, setting, and data sources

A retrospective drug utilization study was conducted using data from the pregnancy subset of the University of Groningen IADB.nl prescription database in the Netherlands (IADB.nl 2020). Between 1994 and 2020, this longitudinal database collected 2,755,976 prescriptions for more than 1.2 million people in the northeastern Netherlands. These prescribed medications were collected from approximately 120 community pharmacies. The database is representative of the Dutch population as a whole (Schirm et al. 2004; Visser et al. 2013).

Medications retrieved during hospitalization or over-the-counter medicines are not recorded in the IADB.nl database. Each prescription contains information on a drug dispensing date, quantity of a drug dispensed, dose regimen, duration of a drug prescription, prescribing physician, and the Anatomical Therapeutic Chemical (ATC) code. The ATC code in the IADB.nl database refers to ATC/DDD index (WHOCC 2020). Data of date of birth and gender are stored, and a unique anonymous identifier is assigned to each patient.

The IADB.nl pregnancy database is a subset of the main IADB.nl longitudinal prescription database. Prescription data in this database is expanding over time because data from more pharmacies is included each year. Besides, the population pyramid of males and females between data in the IADB.nl and Statistics Netherlands (CBS) from 1999 forward was in accordance (Sediq et al. 2018). Until now, the data from IADB.nl remains representative of the general population in the Netherlands. An estimated 65% of children in the main IADB database could be linked accurately to their mothers using a validated method based on the address code (Schirm and Tobi 2004). This linkage resulted in 65,967 pairs of mothers and children in this subset (1994–2020). Since the actual pregnancy duration for each mother is unknown, data on the child’s birthdate is used to determine the “theoretical conception date (TCD)” as the birthdate minus 273 days. In our study, the theoretical length of pregnancy is standardized as 39 weeks (9 months or 13 weeks of each trimester), in line with earlier publications (Schirm et al. 2004).

### Study population

#### Inclusion criteria

All singleton pregnancies of mothers with a delivery date between January 1, 2000, and December 31, 2019, in the pregnancy database of the IADB.nl during the entire observation period from six months before conception date and during pregnancy (±15 months before liveborn delivery date) were included in this study.

#### Prescription data collection

For our analyses, we collected all prescriptions, dispensed between two periods of observation (within six months before the theoretical conception date and during pregnancy period of 39 weeks), for any of the following psychotropic drugs: antipsychotics (+ lithium) (ATC N05A), anxiolytics (ATC N05B), sedative/hypnotics (ATC N05C), antidepressants (ATC N06A), and psychostimulants (ATC N06B).

### Data analysis

We distinguished prescribing patterns of psychotropic medication based on the time windows of drug exposure: before pregnancy only, during pregnancy only, and both before and during pregnancy. Continuation rate (CR) was defined as proportion of singleton pregnancies when mothers filled ≥ one prescription before theoretical conception date (TCD) and ≥ one prescription during pregnancy per 1000 total population of singleton pregnancies. Initiation rate (IR) was defined as proportion of mothers who filled ≥ one prescription anytime during pregnancy and no prescription before TCD per 1000 total population. Discontinuation rate (DR) was defined as proportion of mothers who filled ≥ one prescription only before TCD and no prescription anytime during pregnancy per 1000 total population. Total exposure rate (TER) was the addition of CR and IR to represent the total exposure to psychotropic medication during pregnancy regardless of continued or initiated use. All exposure rates (CR, IR, DR, and TER) of psychotropic treatments were calculated for each decade with the total singleton pregnancies population as the denominator. The top five prescribed psychotropic drugs were listed based on the highest TER per decade. The rankings were compared to observe any difference in the preference of drug choices prescribed between both decades.

In the case that a psychotropic drug is either continued or initiated during pregnancy, it is considered an “accepted treatment” in contrast to discontinued treatments. Acceptance rate or “percentage of accepted treatment (PAT),” defined as [TER/(TER + DR)] × 100%, was calculated for various classes of psychotropics as well as individual drugs for both decades. Only psychotropics with an exposure rate ≥ 0.10 per 1000 pregnancies are included in the analyses on the individual drug.

Student’s *t* test and Pearson’s X2 were used to assess the study population’s characteristics (age of women at delivery), exposure rates (CR, IR, DR, and TER), and PAT for each psychotropic between both decades, as appropriate. A significance level cut-off was a two-tailed *p* value < 0.05. All statistical analyses were conducted using IBM SPSS Statistics version 27 (Chicago, IL, USA).

## Results

### Characteristics of the study population

7,582 singleton pregnancies (decade 1: 3,218; decade 2: 4,364) from a total population of 59,171 singleton pregnancies (decade 1: 27,185; decade 2: 31,986) were identified in the IADB.nl pregnancy database (Table 1). We found an increased rate of singleton pregnancies exposed to all psychotropics (118.4 to 136.5 per 1000 singleton pregnancies), to any antipsychotics (4.9 to 9.8) and any antidepressants (44.7 to 59.6) anytime before and/or during pregnancy between both decades. The age of mothers at delivery before and after stratified by time windows of drug exposure and psychotropic classes exposed were comparable between both decades (Table 1).

**Table 1:** Characteristics of the Dutch singleton pregnancies included in the study

Exposure to psychotropic drugs	Decade 1 (2000–2009)			Decade 2 (2010–2019)		
	*n*	Exposure rate (ER)	Age of mother at delivery (mean ± SD)	*n*	Exposure rate (ER) *p* value^d^	Age of mother at delivery (mean ± SD) *p* value^e^
Total singleton pregnancies exposed	3,218	118.4	30.6 ± 5.1	4,364	136.5^***^	30.7 ± 4.9
*a. Time window of drug use*						
Before pregnancy only^a^	1,644	60.5	30.2 ± 5.0	1,779	55.6^*^	30.1 ± 5.0
During pregnancy only^b^	807	29.7	30.6 ± 5.1	1,080	33.8^**^	30.5 ± 5.0
Before and during pregnancy^c^	767	28.2	31.9 ± 5.0	1.505	47.1^***^	31.6 ± 4.8
*b. Psychotropic class exposed*						
Antipsychotic users	132	4.9	30.9 ± 6.1	312	9.8^***^	30.9 ± 5.4
Anxiolytic users	1,154	42.5	30.3 ± 5.1	1,052	32.9^***^	30.6 ± 4.7
Sedative/hypnotic users	691	25.4	30.9 ± 5.0	911	28.5^*^	30.7 ± 5.2
Antidepressant users	1,216	44.7	30.9 ± 5.1	1,906	59.6^***^	30.7 ± 4.8
Psychostimulant users	25	0.9	27.0 ± 5.1	183	5.7^***^	28.8 ± 4.9

^a^Discontinued users; ^b^initiated users; ^c^continued users; ^d^Pearson’s *X*^2^ test, significant at ^*^p < 0.05; ^**^p < 0.01; ^***^p < 0.001; ^e^Student’s *t*-test (a significance level of 0.05); *ER*, number of exposed pregnancies per 1000 singleton pregnancies. Total population of singleton pregnancies in decade 1 (*n* = 27,185) and decade 2 (*n* = 31,986)

### Most commonly prescribed psychotropic drugs during pregnancy between the two decades

Over the past two decades, the top five most prescribed psychotropic drugs were predominantly antidepressants belonging to the SSRIs and benzodiazepines subclasses (Table 2). Temazepam, a sedative/hypnotic drug, remained the most prescribed during pregnancy for two decades. Oxazepam and diazepam were the most prescribed anxiolytic drugs in decade 1, but only oxazepam appeared in the top five drugs in decade 2. The most prescribed antidepressants in decade 1 were paroxetine and fluoxetine, but in decade 2, the antidepressant preference shifted to citalopram, paroxetine, and sertraline.

**Table 2:** A top-five commonly prescribed psychotropics during pregnancy between the two decades

Decade 1 (2000–2009)				Decade 2 (2010–2019)			
Psychotropic drug	Psychotropic class	*n*	Total exposure rate (TER)^a^	Psychotropic drug	Psychotropic class	*n*	Total exposure rate (TER)
1. Temazepam, BDZ	Sedative/hypnotic	320	11.8	1. Temazepam, BDZ	Sedative/hypnotic	439	13.7
2. Oxazepam, BDZ	Anxiolytic	257	9.5	2. Citalopram, SSRI	Antidepressant	319	10.0
3. Paroxetine, SSRI	Antidepressant	220	8.1	3. Oxazepam, BDZ	Anxiolytic	313	9.8
4. Diazepam, BDZ	Anxiolytic	135	5.0	4. Paroxetine, SSRI	Antidepressant	210	6.6
5. Fluoxetine, SSRI	Antidepressant	88	3.2	5. Sertraline, SSRI	Antidepressant	210	6.6

^a^Total exposure rate of exposed pregnancies per 1000 singleton pregnancies; *BDZ*, benzodiazepine; *SSRI*, selective serotonin reuptake inhibitor. Total population of singleton pregnancies in decade 1 (*n* = 27,185) and decade 2 (*n* = 31,986)

### Exposure rates per 1000 singleton pregnancies of five psychotropic classes before and during pregnancy in both decades

Both continuation rate (CR: 28.2 vs. 47.1) and initiation rate (IR: 29.7 vs. 33.8) of all classes of psychotropics showed an increasing trend between decades resulting in an increased total exposure rate (TER: 57.9 to 80.8) (Table 3). In contrast to CR and IR, DR of all psychotropic classes decreased (DR: 60.5 to 55.6) between decades.

**Table 3:** Comparisons in the exposure rates^a^ per 1000 singleton pregnancies of psychotropic drugs over the last two decades (decade 1 2000–2009; decade 2 2010–2019)

Psychotropic drugs exposed before and/or during pregnancy	Decade 1		Decade 2		*p* value^f^	Decade 1		Decade 2		*p* value	Decade 1		Decade 2		*p* value	Decade 1		Decade 2		*p* value
	*n*	TER^b^	*n*	TER		*n*	CR^c^	*n*	CR		*n*	IR^d^	*n*	IR		*n*	DR^e^	*n*	DR	
Combined of all five classes of psychotropics	1,574	57.9	2,585	80.8	***	767	28.2	1,505	47.1	***	807	29.7	1,080	33.8	**	1,644	60.5	1,779	55.6	*
*N05A antipsychotic* *(+ lithium)*	89	3.3	217	6.8	***	43	1.6	142	4.4	***	46	1.7	75	2.3		43	1.6	95	3.0	**
N05AD01 haloperidol	18	0.7	46	1.4	**	11	0.4	14	0.4		7	0.3	32	1.0	***	3	0.1	6	.2	
N05AH03 olanzapine	4	0.1	25	0.8	***	2	0.1	16	0.5	**	2	0.1	9	0.3		7	0.3	10	0.3	
N05AH04 quetiapine	10	0.4	94	3.1	***	7	0.3	70	2.2	***	3	0.1	24	0.8	***	9	0.3	50	1.6	***
N05AX08 risperidone	9	0.3	15	0.5		7	0.3	13	0.4		2	0.1	2	0.1		7	0.3	10	0.3	
N05AN01 lithium	12	0.4	16	0.5		9	0.3	13	0.4		3	0.1	3	0.1		5	0.2	3	0.1	
Others	36	1.3	21	0.7	**	7	0.3	16	0.5		29	1.1	5	0.2	***	12	0.4	16	0.5	
*N05B anxiolytic*	468	17.2	466	14.6	*	164	6.0	181	5.7		304	11.2	285	8.9	**	686	25.2	586	18.3	***
N05BA01 diazepam	135	5.0	67	2.1	***	38	1.4	29	0.9		97	3.6	38	1.2	***	293	10.8	143	4.5	***
N05BA04 oxazepam	257	9.5	313	9.8		93	3.4	100	3.1		164	6.0	213	6.7		306	11.3	367	11.5	
N05BA06 lorazepam	16	0.6	27	0.8		2	0.1	13	0.4	*	14	0.5	14	0.4		10	0.4	28	0.9	*
N05BA12 alprazolam	25	0.9	32	1.0		15	0.6	26	0.8		10	0.4	6	0.2		45	1.7	27	0.8	**
Others	35	1.3	27	0.8		16	0.6	13	0.4		19	0.7	14	0.4		32	1.2	21	0.7	*
N05C sedative/hypnotic	379	13.9	527	16.5	*	68	2.5	117	3.7	*	311	11.4	410	12.8		312	11.5	384	12.0	
N05CD06 lormetazepam	13	0.5	7	0.2		7	0.3	4	0.1		6	0.2	3	0.1		29	1.1	15	0.5	**
N05CD07 temazepam	320	11.8	439	13.7	*	42	1.5	79	2.5	*	278	10.2	360	11.3		207	7.6	249	7.8	
N05CF01 zopiclone	9	0.3	21	0.7		7	0.3	11	0.3		2	0.1	10	0.3	*	15	0.6	33	1.0	*
N05CF02 zolpidem	10	0.4	37	1.2	**	5	0.2	13	0.4		5	0.2	24	0.8	**	28	1.0	29	0.9	
Others	27	1.0	23	0.7		7	0.3	10	0.3		20	0.7	13	0.4		33	1.2	58	1.8	
N06A antidepressant	624	23.0	1,298	40.6	***	482	17.7	998	31.2	***	142	5.2	300	9.4	***	592	21.8	608	19.0	*
N06AA04 clomipramine	33	1.2	33	1.0		21	0.8	31	1.0		12	0.4	2	0.1	**	16	0.6	6	0.2	*
N06AA09 amitriptyline	63	2.3	97	3.0		42	1.5	69	2.2		21	0.8	28	0.9		93	3.4	127	4.0	
N06AB03 fluoxetine	88	3.2	96	3.0		59	2.2	79	2.5		29	1.1	17	0.5	*	56	2.1	38	1.2	**
N06AB04 citalopram	62	2.3	319	10.0	***	48	1.8	250	7.8	***	14	0.5	69	2.2	***	61	2.2	110	3.4	**
N06AB05 paroxetine	220	8.1	210	6.6	*	181	6.7	184	5.8		39	1.4	26	0.8	*	193	7.1	79	2.5	***
N06AB06 sertraline	17	0.6	210	6.6	***	15	0.6	118	3.7	***	2	0.1	92	2.9	***	30	1.1	44	1.4	
N06AB08 fluvoxamine	36	1.3	19	0.6	**	31	1.1	16	0.5	**	5	0.2	3	0.1		11	0.4	7	0.2	
N06AB10 escitalopram	6	0.2	86	2.7	***	5	0.2	72	2.3	***	1	0.04	14	0.4	**	9	0.3	38	1.2	***
N06AX11 mirtazapine	21	0.8	49	1.5	**	17	0.6	32	1.0		4	0.1	17	0.5	*	34	1.3	45	1.4	
Others	78	2.9	179	5.6	***	63	2.3	147	4.6	***	15	0.6	32	1.0		89	3.3	114	3.6	
N06B psychostimulant	14	0.5	77	2.4	***	10	0.4	67	2.1	***	4	0.1	10	0.3		11	0.4	106	3.3	***
N06BA04 methylphenidate	13	0.5	58	1.8	***	9	0.3	52	1.6	***	4	0.1	6	0.2		8	0.3	88	2.8	***
Others	1	0.04	19	0.6	***	1	0.04	15	0.5	**	0	0	4	0.1		3	0.1	18	0.6	**

^a^Only psychotropic drugs with exposure rates ≥ 0.10 in both decades were displayed; ^b^total exposure rate per 1000 singleton pregnancies; ^c^*CR*, continuation rate per 1000 singleton pregnancies; ^d^*IR*, initiation rate per 1000 singleton pregnancy population; ^e^*DR*, discontinuation rate per 1000 singleton pregnancies; ^f^Pearson’s *X*2 test, significant at ^*^p < 0.05; ^**^p < 0.01; ^***^p < 0.001. Total population of singleton pregnancies in decade 1 (*n* = 27,185) and decade 2 (*n* = 31,986)

In Table 3, we compared the exposure rates for each class of psychotropics between decades. The TER of antipsychotics increased in decade 2 with the most frequently prescribed antipsychotic drugs being haloperidol in decade 1 and quetiapine in decade 2. Antipsychotic drugs with an increased exposure rate during pregnancy were quetiapine (both CR and IR), haloperidol (only IR), and olanzapine (only CR). The DR of antipsychotic drugs was comparable between decades, except for quetiapine (increased from 0.3 to 1.6).

Treatment with anxiolytics and sedatives/hypnotics was more often initiated during pregnancy than continued before pregnancy (Table 3). The exposure rate of oxazepam and temazepam was the highest among continued, initiated, and discontinued users in both decades. The IR of z-drugs like zolpidem and zopiclone was much lower than the IR of temazepam in both decades.

Both CR and IR of antidepressants were almost twice higher in decade 2, resulting in an increase in its TER during pregnancy (Table 3). Paroxetine was the most prescribed antidepressant during pregnancy in decade 1. However, in decade 2, citalopram became the most continued antidepressant drug. Citalopram and sertraline became the most prescribed antidepressants in continued users due to increased CR and IR. An increasing trend of the CR and DR of psychostimulants, including methylphenidate, was also observed over decades.

### Acceptance rates of different psychotropic treatments used by pregnant women in both decades

**Fig. 1:**
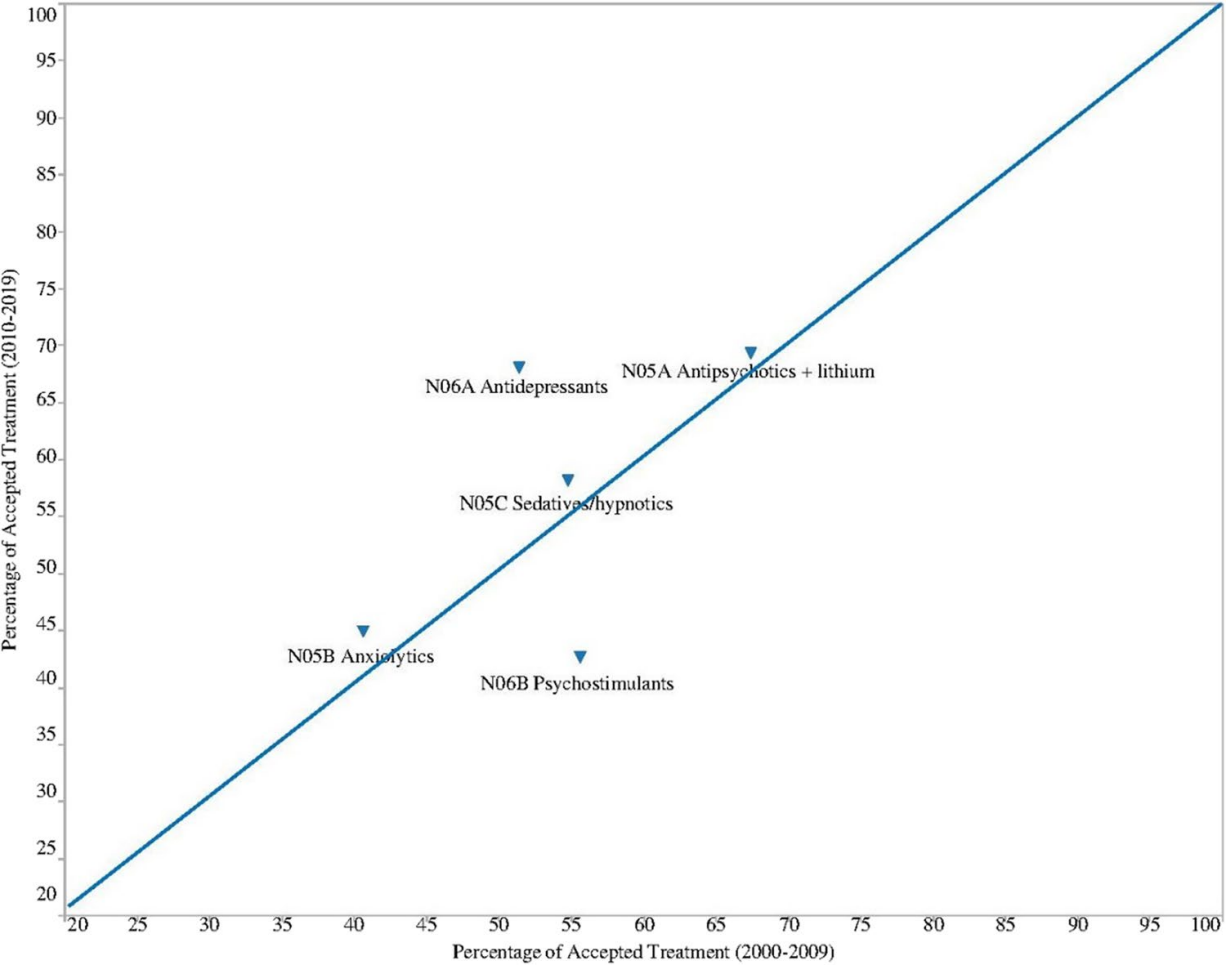
The *XY* plot^a^ of percentage of accepted treatment (PAT) of five classes of psychotropics compared between the last two decades. ^a^*X*-axis = PAT in decade 1 (2000–2009); *Y*-axis = PAT in decade 2 (2010–2019). Psychotropic classes with a higher PAT in decade 2 are above the diagonal line and those with a lower PAT in decade 2 are below the diagonal

**Fig. 2:**
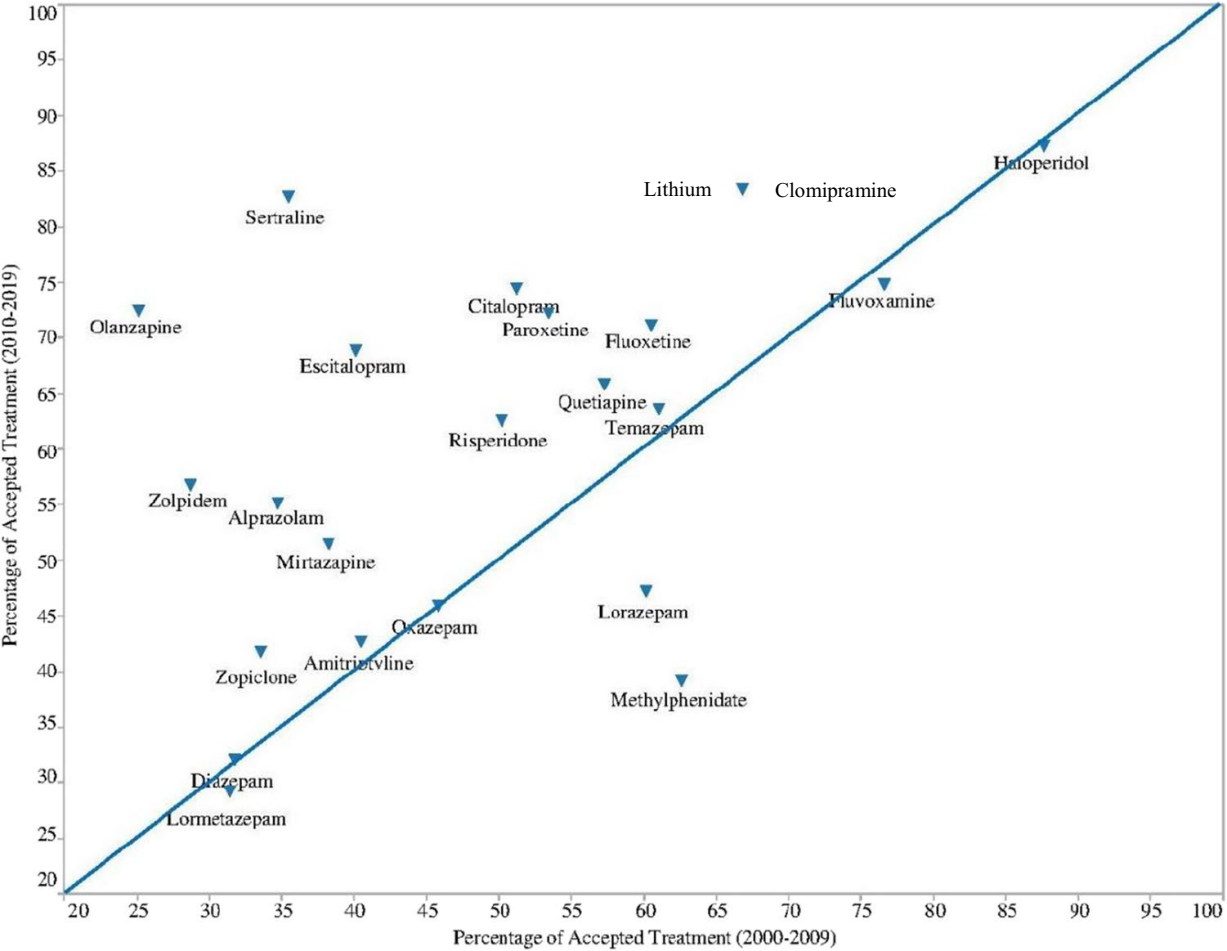
The *XY* plot^a^ of percentage of accepted treatment (PAT) of individual psychotropic drugs compared over the past two decades^b^. ^a^*X*-axis = PAT in decade 1 (2000–2009); *Y*-axis = PAT in decade 2 (2010–2019). Drugs with a higher PAT in decade 2 are above the diagonal line and those with a lower PAT in decade 2 are below the diagonal. ^b^Positions of lithium and clomipramine are overlapping due to the same value of PAT in both decades

The change in the percentage of accepted treatment using psychotropics between decades was illustrated in Fig. 1 (PAT of psychotropic classes) and Fig. 2 (PAT of individual drugs). Psychotropic classes and individual drugs with a higher PAT in decade 2 than in decade 1 were plotted above the diagonal line and vice versa. In Fig. 1, we observed an increased PAT of antidepressant class and a decreased PAT for psychostimulant class in decade 2. The PAT for other classes (antipsychotic, sedative/hypnotic, and anxiolytic) remained comparable between decades.

Individual drugs with an increase of PAT in decade 2, including olanzapine (all atypical APs), lithium (mood stabilizer), sertraline, citalopram, paroxetine, and escitalopram (all SSRIs). In contrast, a lower PAT of lorazepam (anxiolytic) and methylphenidate (psychostimulant) was seen in decade 2. The comparison of acceptance rates of psychotropic classes and individual drugs between decades was presented in Supplementary 1 Table (S1 Table).

## Discussion

We observed an increasing trend in exposure and acceptance rates of psychotropic drugs from decade 1 (2000–2009) to decade 2 (2010–2019) among Dutch pregnant women. Looking at the exposure pattern at the class level, the most continued drugs among pregnant women are antipsychotics and antidepressants, mainly shifting from haloperidol (typical) and paroxetine (SSRI) in decade 1 to quetiapine (atypical) and citalopram (SSRI) in decade 2. Other findings are discussed in more detail below.

### Comparison of exposure and acceptance rates of individual psychotropic drug use between the past two decades

#### Antipsychotic drugs and lithium

The TER of antipsychotics in the Netherlands doubled over time mainly due to increase both continuation and initiation rates of atypical antipsychotics in pregnancy. This result is in line with a previous study in Denmark (2000–2016) (Damkier et al. 2018). A shifting trend from typical to atypical antipsychotics used during pregnancy was observed except for haloperidol, where its exposure rate was consistent for two decades. The acceptance rate of haloperidol treatment also remained high in pregnancy between decades. The reason for this potentially because the continued use of haloperidol is recommended during pregnancy as the consensus between the Dutch Association for Obstetrics and Gynecology (NVOG), the Dutch Association for Pediatrics (NVK), and the Dutch Association for Psychiatry (NVvP) (Federatie Medisch Specialisten 2020a). The use of haloperidol during the first and second trimesters is considered safe concerning the risk of gestational diabetes and potential teratogenic effects (Federatie Medisch Specialisten 2020a). Treatments with atypical drugs, e.g., olanzapine, quetiapine, and risperidone, received a higher acceptance rate in decade 2, meaning that haloperidol is not always the first choice of antipsychotic given during pregnancy.

The indications for which antipsychotics are prescribed belong to the more severe psychiatric illnesses such as psychosis and bipolar disorder. Due to this, the continuation of a well-adjusted antipsychotic treatment becomes more important than discontinuing its use during pregnancy (Andrade 2021). Considering that some pregnant women will be exposed to antipsychotic(s), the guidelines mention that the use of antipsychotic drugs is relatively safe concerning the development of congenital malformations, pregnancy, and birth complications (Federatie Medisch Specialisten 2020a). Furthermore, antipsychotics do not seem to increase the risk of neurodevelopmental disorders in offspring like Attention Deficit-Hyperactivity Disorder (ADHD) and Autism Spectrum Disorder (ASD) (Wang et al. 2021; Straub et al. 2022). Although the knowledge about the long-term effects of antipsychotics is limited, NVOG recommends continuing its use during pregnancy since the benefits of a well-treated psychotic disorder may outweigh its potential risk to the mother and fetus (Federatie Medisch Specialisten 2020a).

The use of lithium as the first-line treatment for bipolar disorders was relatively constant over time. NVOG recommends continuing lithium monotherapy as the drug of choice during pregnancy, with the lowest effective dose and frequently checked blood level (Federatie Medisch Specialisten 2020a). Although a potential risk of congenital heart defects has been described (Patorno et al. 2017), lithium was observed to be prescribed frequently and consistently during pregnancy in our study. This trend reflects that a safe alternative to lithium is lacking.

#### Anxiolytic drugs

A decrease in the exposure rate of anxiolytics was observed during pregnancy, especially diazepam, which was less often initiated over time. The acceptance rate of anxiolytic treatment over decades was also consistently low for diazepam, oxazepam, lorazepam, and alprazolam. This finding aligned with the guidelines of NVOG that benzodiazepine should be prescribed for a short period during pregnancy. The use of anxiolytics is not recommended for long-term use, higher dose, or combination with other medication as these factors may lead to neonatal withdrawal symptoms after birth and carry a risk of drug addiction or dependence (Bellantuono et al. 2019; Federatie Medisch Specialisten 2020b).

#### Sedative/hypnotic drugs

The bigger proportion of pregnant women who received sedatives/hypnotics during pregnancy was predominantly initiated users. This finding was in line with the NVOG recommendation, in which benzodiazepines are indicated only for acute relief of anxiety and short-term treatment of sleep problems. Therefore, these drugs are only prescribed when necessary (Federatie Medisch Specialisten 2020b). An increase in exposure rate to sedatives/hypnotics mainly resulted from an increase in the continuation and initiation use of temazepam. Prescription of temazepam became the most frequently given during pregnancy, and its acceptance rate increased over time. We assumed this trend might occur due to temazepam’s short half-life, no active metabolite, and no dose accumulated after repeated administration (Bellantuono et al. 2019; Edinoff et al. 2021).

#### Antidepressant drugs

We observed a two-fold increase in the TER of antidepressants due to a doubled increase in its continuation and initiation rate. Over decades, SSRI drugs were more prescribed among pregnant Dutch women than non-SSRIs, such as TCAs. This finding was in line with previous reports regarding the increased trend in the use of SSRIs in the Netherlands (1995–2004 and 1999–2014) (Bakker et al. 2008; Molenaar et al. 2020). The most commonly prescribed antidepressants reported in the United States (2006–2011) during pregnancy were SSRI drugs (Hanley and Mintzes 2014). The use of SSRIs among pregnant women in France and New Zealand was also more frequent than non-SSRI antidepressants (Bénard-Laribière et al. 2018; Donald et al. 2021). The current guidelines recommend continuing any SSRI prescribed before conception (Federatie Medisch Specialisten 2020c). Expanding knowledge from reports on safe use of antidepressants in pregnancy may influence why the prescribing trend of SSRI over decades, either as continued treatment, initiated, or both.

When looking at the individual drugs, the exposure rate of citalopram, sertraline, and escitalopram increased highly in decade 2. These SSRIs are considered safer options compared to paroxetine and are also recommended as the first choice of SSRI in the guidelines (Federatie Medisch Specialisten 2020c). The exposure rate of paroxetine decreased in decade 2 and this trend aligned with the NVOG which recommends switching from paroxetine to other SSRIs, e.g., sertraline and citalopram if necessary. The guidelines mention that switching of paroxetine should take place before conception to limit the risk of congenital cardiac malformations (Federatie Medisch Specialisten 2020c). Although the use of SSRIs is considered safe, close monitoring pre- and postnatally is always advised to anticipate the potential risks of SSRI use in newborns, such as preterm birth, congenital heart defects, and persistent pulmonary hypertension (Pedersen et al. 2009; Jong et al. 2012; Huybrechts et al. 2014). Nevertheless, studies have reported that prenatal exposure to SSRIs in pregnancy was not linked with ADHD risk in offspring (Laugesen et al. 2013; Lupattelli et al. 2021; Hartwig et al. 2022).

#### Psychostimulant drugs

In the Netherlands, methylphenidate has been recommended as the first-line treatment for ADHD in adults by the Dutch Association for Psychiatry (NVvP) since 2015. Before this period, methylphenidate was not approved for use by adults (Federatie Medisch Specialisten 2020d). After this recommendation was introduced, we observed that the exposure to psychostimulants, mostly methylphenidate, increased during pregnancy. This change was mainly due to a high increase in its continuation rate in decade 2. However, the acceptance rate of methylphenidate during pregnancy remained low in the last decade. Considering the approval of this drug was less than ten years, the acceptance rate of methylphenidate during pregnancy remained low, resulting in more discontinued users compared to continued and initiated users in decade 2. This finding is in line with the recommendations that do not advise the use methylphenidate, especially in the second and third trimesters due to a limited report of its safe use for the mother and unborn child (Bijwerkingen Centrum Lareb 2020; Farmacotherapeutisch Kompas 2020).

### Limitations and strengths

Our study is subject to some considerations. The pregnancy IADB.nl subset only captures the prescription information of mothers and children pairs. Therefore, pregnant and exposed women with other pregnancy outcomes (stillborn, miscarriages, or terminations) were not included in our analysis. The exposure rates of psychotropic medications are measured via information about the dispensing of drugs in our prescription database and may not directly measure the actual use of these drug(s) by pregnant women. Furthermore, we defined exposure as receiving one or more dispensing of the drug of interest. Two or more dispensing could indicate exposure to a psychotropic treatment with more certainty. However, one dispensing could already mean an increased risk in exposed women. In our study population, 27.6% and 17.3% of pregnancies received only one dispensing of a psychotropic drug before or during pregnancy, respectively.

For the continuation rate of a psychotropic, we did not consider the time interval between two dispensings, as well as the duration of each psychotropic treatment. Finally, the pregnancy starting date was based on the theoretical conception date (39 weeks before the child's birthdate), which may lead to misclassification of the exposure window to a psychotropic drug since not all livebirths were delivered precisely within the same period (39th week).

The strengths of this study presented the changing of actual prescribing trends of all five classes of psychotropics (antipsychotic, anxiolytic, sedative/hypnotic, antidepressant, and psychostimulant) by showing their exposure rates (TER, CR, IR, and DR) during the past twenty years. The “acceptance rate (%)” we presented mainly reflected the acceptance by the prescribing physician. A prescriber, of course, could have discussed the decision to continue a psychotropic treatment with a patient. Hence, the acceptance rate used in this study could also reflect the doctor's and patient’s acceptance.

An increased trend in the exposure rate of psychotropics during pregnancy, especially the antidepressant and antipsychotic classes in decade 2 and this change could be influenced by some factors, e.g., an increased prevalence of psychiatric illnesses in women of reproductive age population, more experiences reported regarding the safe use of psychotropics, recommendations to continue the use of psychotropic treatment despite a woman being pregnant, and a higher acceptance of prescribers to continue prescribing a psychotropic in pregnancy. Furthermore, results in this study are informative for clinicians, pharmacists, and all involved caregivers regarding the most recent and actual prescribing trend of five classes of psychotropics in clinical practice and the way this relates to the current guidelines concerning the advice use of psychotropics in pregnancy. It should be noted that the decision to continue, discontinue, or initiate the use of psychotropics during pregnancy should be decided on a case-by-case basis, for example, the difference in the severity of psychiatric illnesses. Further study is warranted to investigate potential switching patterns and possible dosage adjustments of psychotropic drug use before pregnancy and between trimesters of pregnancy, specifically for drugs with potential risks in pregnancy, e.g., paroxetine, lithium, and the like.

## Conclusion

The actual prescribing trends, especially antipsychotics and antidepressants among the Dutch pregnancy population, have changed over time. During decade 2, the exposure and acceptance rates of certain antipsychotics and antidepressants significantly increased during pregnancy compared to the preceding decade. It seems that the choice of psychotropic drugs for pregnant women in the Netherlands over decades was aligned with the recommendation from NVOG, the Dutch Association of Obstetrics and Gynecology. Findings in our study suggest a prescribing trend toward a well-considered preference for safer psychotropic treatment during pregnancy over the past years.

## Supplementary Information

ESM 1 (DOCX 17.1 kb)

## Data Availability

The dataset for this manuscript is not publicly available due to the IADB data protection policy. Request to access the database should be directed to the corresponding author upon reasonable request.

## Code availability

Programming code is available on request.
