# Supplementary material for: Exposure to psychotropic drugs before and during pregnancy: what has changed over the last two decades?
Source: Arch Womens Ment Health. 2023 Jan 14;26(1):39–48. doi: 10.1007/s00737-023-01290-8 (PMC9908723; doi:10.1007/s00737-023-01290-8)
Supplement: Supplementary file 1 — (DOCX 17.1 kb) [file 737_2023_1290_MOESM1_ESM.docx]

**Supplementary 1 Table.** Comparison of the acceptance rates of different psychotropic classes and individual drugs over the past two decades

| Psychotropics used by pregnant women^a^ | Percentage of Accepted Treatment (PAT) | |
| --- | --- | --- |
|  | Decade 1 (2000-2009) | Decade 2 (2010-2019) |
| Combined classes of psychotropics | 48.9 | 59.2*** |
| *N05A Antipsychotics (+ lithium)* | 67.3 | 69.4 |
| N05AD01 Haloperidol | 87.5 | 87.5 |
| N05AH03 Olanzapine | 25.0 | 72.7*** |
| N05AH04 Quetiapine | 57.1 | 66.0*** |
| N05AX08 Risperidone | 50.0 | 62.5*** |
| N05AN01 Lithium | 66.7 | 83.3*** |
| *N05B Anxiolytics* | 40.6 | 44.4 |
| N05BA01 Diazepam | 31.6 | 31.8 |
| N05BA04 Oxazepam | 45.7 | 46.0 |
| N05BA06 Lorazepam | 60.0 | 47.1*** |
| N05BA12 Alprazolam | 34.6 | 55.6*** |
| *N05C Sedatives/hypnotics* | 54.7 | 57.9 |
| N05CD06 Lormetazepam | 31.3 | 28.6 |
| N05CD07 Temazepam | 60.8 | 63.7 |
| N05CF01 Zopiclone | 33.3 | 41.2*** |
| N05CF02 Zolpidem | 28.6 | 57.1*** |
| *N06A Antidepressants* | 51.3 | 68.1*** |
| N06AA04 Clomipramine | 66.7 | 83.3*** |
| N06AA09 Amitriptyline | 40.4 | 42.9 |
| N06AB03 Fluoxetine | 60.4 | 71.4*** |
| N06AB04 Citalopram | 51.1 | 74.6*** |
| N06AB05 Paroxetine | 53.3 | 72.5*** |
| N06AB06 Sertraline | 35.3 | 82.5*** |
| N06AB08 Fluvoxamine | 76.5 | 75.0 |
| N06AB10 Escitalopram | 40.0 | 69.2*** |
| N06AX11 Mirtazapine | 38.1 | 51.7*** |
| *N06B Psychostimulants* | 55.6 | 42.1*** |
| N06BA04 Methylphenidate | 62.5 | 39.1*** |

^a^only psychotropic drugs with exposure rates ≥ 0.10 in both decades were displayed

^***^significant at p< 0.001
